# Peptide IDR-1002 Inhibits NF-κB Nuclear Translocation by Inhibition of IκBα Degradation and Activates p38/ERK1/2–MSK1-Dependent CREB Phosphorylation in Macrophages Stimulated with Lipopolysaccharide

**DOI:** 10.3389/fimmu.2016.00533

**Published:** 2016-11-25

**Authors:** Alejandro Huante-Mendoza, Octavio Silva-García, Javier Oviedo-Boyso, Robert E. W. Hancock, Víctor M. Baizabal-Aguirre

**Affiliations:** ^1^Laboratory of Molecular Immunology and Signal Transduction, Facultad de Medicina Veterinaria y Zootecnia, Centro Multidisciplinario de Estudios en Biotecnología, Universidad Michoacana de San Nicolás de Hidalgo, Morelia, Michoacán, México; ^2^Department of Microbiology and Immunology, Centre for Microbial Diseases and Immunity Research, University of British Columbia, Vancouver, BC, Canada

**Keywords:** IDR, peptides, inflammation, macrophages, IκBα, NF-κB, CREB, TNF-α

## Abstract

The inflammatory response is a critical molecular defense mechanism of the innate immune system that mediates the elimination of disease-causing bacteria. Repair of the damaged tissue, and the reestablishment of homeostasis, must be accomplished after elimination of the pathogen. The innate defense regulators (IDRs) are short cationic peptides that mimic natural host defense peptides and are effective in eliminating pathogens by enhancing the activity of the immune system while controlling the inflammatory response. Although the role of different IDRs as modulators of inflammation has been reported, there have been only limited studies of the signaling molecules regulated by this type of peptide. The present study investigated the effect of IDR-1002 on nuclear factor κB (NF-κB) and cAMP-response element-binding protein (CREB) transcription factors that are responsible for triggering and controlling inflammation, respectively, in macrophages. We found that TNF-α and COX-2 expression, IκBα phosphorylation, and NF-κB nuclear translocation were strongly inhibited in macrophages pre-incubated with IDR-1002 and then stimulated with lipopolysaccharide (LPS). IDR-1002 also increased CREB phosphorylation at Ser133 *via* activation of the p38/ERK1/2–MSK1 signaling pathways without detectable expression of the cytokines IL-4, IL-10, and IL-13 involved is suppressing inflammation or alternative activation. Transcriptional activation of NF-κB and CREB is known to require interaction with the transcriptional coactivator CREB-binding protein (CBP). To test for CBP–NF-κB and CBP–CREB complex formation, we performed co-immunoprecipitation assays. These assays showed that IDR-1002 inhibited the interaction between CBP and NF-κB in macrophages stimulated with LPS, which might explain the inhibition of TNF-α and COX-2 expression. Furthermore, the complex between CBP and CREB in macrophages stimulated with IDR-1002 was also inhibited, which might explain why IDR-1002 did not lead to expression of IL-4, IL-10, and IL-13, even though it induced an increase in phospho-CREB relative abundance. In conclusion, our results indicated that IDR-1002 has a dual effect. On one hand, it inhibited NF-κB nuclear translocation through a mechanism that involved inhibition of IκBα phosphorylation, and on the other, it activated a protein kinase signaling cascade that phosphorylated CREB to selectively influence cytokine gene expression. Based on these results, we think IDR-1002 could be a potential good biopharmaceutical candidate to control inflammation.

## Introduction

The inflammatory response is one of the main defense mechanisms of the innate immune system that is activated by host infection with microbial pathogens or tissue damage. This response is fundamental to identify and neutralize the causative agent and restore tissue homeostasis (1). However, in some cases, inflammation can be detrimental, especially when the control or resolution mechanisms fail, leading to chronic inflammatory conditions such as arteriosclerosis, insulin resistance, rheumatoid arthritis, and Alzheimer’s disease (2).

The balance between the inflammatory response and its resolution is maintained in part by the activity of two transcription factors with opposite roles, the nuclear factor κB (NF-κB) and the cAMP-response element-binding protein (CREB). Activation of NF-κB induces expression of pro-inflammatory genes (e.g., TNF-α, IL-6, IL-8, and COX-2), while activation of CREB leads to gene expression of cytokines that have an anti-inflammatory role (e.g., IL-4, IL-10, and IL-13) (3, 4). NF-κB has five subunits but the heterodimer of NF-κB, constituted of p65 and p50 subunits, is one of the most studied active forms of this family of transcription factors (5, 6). When cells are not stimulated, NF-κB is anchored in the cytosol to IκBα protein. After stimulation, IκBα becomes phosphorylated, ubiquitinated, and degraded by the 26S proteosome. Degradation of IκBα releases NF-κB that is translocated to the nucleus where it initiates a complex transcriptional response (7). Once in the nucleus NF-κB can interact with a transcriptional coactivator CREB-binding protein (CBP) and initiate a distinct transcriptional pattern. Activation of NF-κB can also involve phosphorylation of several residues in the transactivation domain of p65 subunit, among which Ser536 is well characterized (8–10).

cAMP-response element-binding protein is a cellular transcription factor that belongs to the basic leucine-zipper domain family of proteins (11). This family is formed by dozens of proteins, including CREB, the cAMP-response element modulator (CREM), and the activating transcription factor-1 (ATF-1). In cells activated by growth factors, hormones, lipopolysaccharide (LPS), and other stimuli (12, 13), CREB is phosphorylated at Ser133 by protein kinase A, protein kinase B (Akt), MSK, or other enzymes (14, 15). Phosphorylation at this residue promotes CREB binding to transcriptional coactivator CBP, which can lead to displacement of NF-κB from the same interaction domain on CBP (16, 17). Formation of CREB–CBP complex promotes the expression of cytokines, typically involved in anti-inflammatory events or alternative activation of monocytes, thereby suppressing expression of pro-inflammatory cytokines activated by the NF-κB–CBP complex (18). Although this mechanism has been widely proposed (19–22), other groups have reported no expression of genes when CREB is phosphorylated at Ser133 downstream of mitogen-activated protein kinase (MAPK) pathways (15, 23, 24). It may imply that control of inflammation by CREB phosphorylation does not necessarily involve the expression of anti-inflammatory genes induced by this transcription factor.

Innate defense regulator (IDR) peptides were designed from the natural theme of cationic host defense peptides (25) as an alternative to traditional anti-inflammatory and antimicrobial drugs. The immunomodulatory effects of IDRs have attracted the attention because of the worldwide problem of multiple antibiotic resistance in bacteria (26–28). In addition to downregulation of the pro-inflammatory response to bacterial ligands, IDRs stimulate protective immunity (enhancing cellular recruitment and promoting differentiation of immune cells) leading to lowering of the bacterial burden without any direct interaction with bacteria (26–28). In addition to their anti-infective and anti-inflammatory effects due to modulation of the immune response, these peptides may also be considered good potential therapeutic candidates to combat chronic inflammation associated with autoimmune diseases (29, 30). One of these peptides, IDR-1002, efficiently enhanced the immune response to eliminate *Staphylococcus aureus* and *Escherichia coli* from infected tissues, even when these bacterial pathogens were multi-resistant to antibiotics (26, 27). Moreover, in synovial fibroblasts, IDR-1002 effectively controlled inflammation by selectively modulating the expression of interleukin (IL)-1Ra and IL-10 and reducing NF-κB p50 nuclear translocation induced by IL-1β (29).

Although several studies have previously reported the potential beneficial effects of IDR-1002, the molecular transduction mechanisms involved in the IDR-1002 activity have not been well described to date (26, 27, 31, 32). Therefore, we focused our attention on the NF-κB and CREB transcription factors, because they are important in triggering and resolving the inflammatory response, respectively. We found that, in RAW264.7 macrophages, stimulated with LPS IDR-1002 was able to inhibit NF-κB nuclear translocation by inhibiting IκBα degradation, leading to a reduction of cycloooxygenase-2 (COX-2), and TNF-α expression. Co-immunoprecipitation analysis of CBP–NF-κB molecular complex showed that IDR-1002 inhibited the interaction between CBP and NF-κB induced by LPS. We also observed that IDR-1002 induced high levels of CREB phosphorylation at Ser133 through activation of p38/ERK1/2–MSK1/2 signaling pathways. Interestingly, we could not detect expression of the anti-inflammatory cytokines IL-4, IL-10, and IL-13, which are known to be regulated by CREB. By applying a similar co-immunoprecipitation analysis, the molecular complex between CBP and CREB was not detected in macrophages stimulated with IDR-1002, which may explain the lack of anti-inflammatory gene expression.

## Materials and Methods

### Media and Chemicals

Innate defense regulator peptides (IDR) 1002 (VQRWLIVWRIRK-NH_2_), HH2 (VQLRIRVAVIRA-NH_2_), and 1 (KSRIVPAIPVSLL-NH_2_) were synthesized using solid phase F-moc chemistry by CPC Scientific, Inc. (Sunnyvale, CA, USA) and were >95% pure. RPMI 1640 cell culture medium, Bradford reagent, bovine serum albumin (BSA), Wortmannin (inhibitor of PI3K), Akt IV (inhibitor of Akt), Rapamycin (inhibitor of p70S6K), SB216763 (inhibitor of GSK3), PD98059 (inhibitor of ERK1/2), and Ro-318220 (inhibitor of MSK1) were purchased from Sigma-Aldrich, Inc. (St. Louis, MO, USA). SB203580 (inhibitor of p38) and SP600125 (inhibitor of JNK) were purchased from Calbiochem (San Diego, CA, USA). Lymphoprep™ was purchased from Axis-Shield PoC AS (Oslo, Norway). Upon arrival, all inhibitors were solubilized in DMSO and stored in aliquots at −20°C. Newborn calf serum was acquired from Hyclone Laboratories, Inc. (South Logan, UT, USA). Penicillin G and streptomycin were purchased from Gibco-BRL (Gaithesburg, MD, USA). EDTA-free protease inhibitor cocktail (50×) was purchased from Roche Applied Science (Manheim, Germany). Antibodies against β-actin (Sc-47778), COX-2 (Sc-1747), GAPDH (Sc-25778), laminin A/C (6215), IκBα (Sc-203), and phospho-IκBα (Sc-52943) for the detection of phosphorylated Ser32 and Ser36, CREB-1 (Sc-186), NF-κB p65 (Sc-8008), CBP (Sc-7300), anti-rabbit IgG-HRP (Sc-2004), rabbit anti-goat IgG-HRP (Sc-2768), and goat anti-mouse IgG-HRP (Sc-2031) were acquired from Santa Cruz Biotechnology, Inc. (Santa Cruz, CA, USA). Antibodies against the phosphorylated forms of CREB at Ser133 (9196), p38 at Thr180/Tyr182 (4511), NF-κB p65 at Ser536 (3033), GSK3α at Ser21 (9316), and GSK3β at Ser9 (9336) were purchased from Cell Signaling Technology (Boston, MA, USA). Antibodies to detect phosphorylated MSK1 at Thr581 (AF2518) were purchased from R&D Systems (Minneapolis, MN, USA). Antibodies to detect the phosphorylated forms of IKKα and β at Thr23 were obtained from Santa Cruz Biotechnology (Cat. Sc-21660, Santa Cruz, CA, USA). Non-fat dry milk was acquired from Bio-Rad (California, CA, USA) and Luminol reagent from Millipore (Billerica, MA, USA).

### Cell Line and Culture Conditions

The mouse leukemic monocyte macrophage cell line RAW 264.7 used was obtained from the American Type Culture Collection (ATCC). This immortalized cell line was grown and maintained in RPMI medium supplemented with 10% FCS, unless otherwise noted.

### Isolation of Human Peripheral Blood Mononuclear Cells

Venous blood (20 ml) from healthy human donors was collected in tubes containing EDTA in accordance with the approval and ethical guidelines of the Universidad Michoacana de San Nicolás de Hidalgo. Blood was diluted in the same volume of RPMI medium. Then, 20 ml of diluted blood was carefully added to a tube containing 10 ml of Lymphoprep™ and centrifuged at 800 × *g* for 20 min at room temperature (23°C). The phase containing peripheral blood mononuclear cells (PBMC) was separated and washed 3× with enough PBS to complete 50 ml for each wash and centrifuged at 250 × *g* for 10 min. After washing, PBMC were resuspended in RPMI medium and seeded in six-well plate at 2 × 10^6^ cells/well. Cells were left resting for at least 3 h before the experiments.

### Gene Knockdown of MSK1 and MSK2

MSK1 and MSK2 gene expression were silenced with siRNA acquired from Santa Cruz Biotechnology (Santa Cruz, CA, USA). The siRNA for MSK1 (sc-35978), MSK2 (sc-75837), and control siRNA (sc-37007) were added to macrophages at the final concentration of 40 nM for 48 h, according to the manufacturer’s instructions. To test for MSK1/MSK2 participation in CREB phosphorylation at Ser133, macrophages were first gene silenced for MSK1 and/or MSK2 and then stimulated with 20 μg/ml of IDR-1002 for 15 min.

### Protein Extraction and Western Blot Analysis

The relative abundance of phosphorylated and non-phosphorylated proteins was evaluated in protein extracts from macrophages grown in six-well culture plates to ~90% confluence before serum starvation for at least 4 h. For each assay, total protein (cytosolic plus nuclear from control and treated cells) was obtained by washing cells with cold PBS (2×) and lysing them with 80 μl of a cold lysis buffer containing 20 mM Tris–HCl pH 7.5, 150 mM NaCl, 1% Igepal CA-930, 10 mM Na-pyrophosphate, and 50 mM NaF, supplemented with 1 mM Na-orthovanadate and 1× protease inhibitor cocktail that was added immediately before use. The lysates were centrifuged at 13,000 × *g* for 20 min at 4°C, and the supernatant was transferred to ice-cold Eppendorf tubes. Nuclear and cytoplasmic fractions were prepared from cells using the NE-PER Reagent kit from Pierce (Rockford, IL, USA), and stored in aliquots at −80°C. Protein concentration was measured by the Bradford method (33) using BSA as standard. Then, 40 μg of protein was separated by electrophoresis in 10% sodium dodecyl sulfate polyacrylamide gels and electroblotted in a wet chamber to 0.45 μm nitrocellulose membrane (Bio-Rad) at 250–300 mA for 1 h. Membranes were then probed with the indicated antibody and the abundance of the phosphorylated and unphosphorylated forms of each protein was detected using the Immobilon Western Chemiluminescent HRP substrate kit from Millipore (Billerica, MA, USA). Membranes were exposed to an X-ray film (Kodak) with two intensifying screens (DuPont) at room temperature (23°C).

### Co-Immunoprecipitation Assays

Macrophages Raw 264.7 were seeded in 10-cm cell plate culture until confluence, left in serum-free media for 4 h, and then stimulated as indicated. For immunoprecipitation technique, we use the kit Pierce™ Protein A/G Magnetic Beads (Thermo Fischer Scientific). In brief, ~1 × 10^7^ cells were lysed in washing/lysis buffer supplemented with 1× protease inhibitor cocktail, and cells debris were collected by centrifugation at 18,000 × *g* for 10 min at 4°C. The supernatant was collected and quantified for protein concentration. Then, 4 μg of each immunoprecipitation antibody (CBP or IκBα) was added to 500 μg of protein extract and the volume adjusted to 500 μl. The mixture was incubated with continuous agitation all night at 4°C. Next day, 25 μg of magnetic beads were added and incubated 1 h in agitation at room temperature (23°C); beads were washed 2× with washing buffer and 1× with water. The target antigen was eluted with lane marker buffer 1× plus 50 mM DTT in the final volume of 100 μl. For immunoblots, 20 μl of target antigen in elution buffer was used and detected as described in the western blot analysis section.

### Cytokine Quantitation

Mouse cytokines, IL-4, IL-6, IL-10, IL-13, TNF-α, and PGE2 levels, were measured by ELISA, according to the manufacturer’s instructions (R&D, Minneapolis, MN, USA).

### Cytokine Array

Macrophages were incubated for 1 h with 20 μg/ml of IDR-1, IDR-HH2, or IDR-1002 and then stimulated for 2–24 h with 10 ng/ml of LPS. Supernatants were collected and cytokines were detected using a mouse cytokine antibody pair-based array spotted on a membrane (Abcam, ab133993) according to the manufacturer’s instructions (Cambridge, UK). Briefly, analysis of cytokines present in the macrophages supernatant is carried out by chemiluminescent western blot assay, using biotinylated detector antibodies and streptavidin HRP. Targets of this ELISA-like array are granulocyte-colony stimulating factor (GCSF), granulocyte-macrophage colony stimulating factor (GM-CSF), IL-2, 3, 4, 5, 6, 9, 10, 12 p40/p70, 12p70, 13, 17, interferon gamma (IFN-γ), monocyte chemoattractant protein 1 and 5 (MCP-1 and MCP-5), regulated on activation normal T cell expressed and secreted (RANTES), stem cell factor (SCF), soluble tumor necrosis receptor factor 1 (sTNFR1), tumor necrosis factor alpha (TNF-α), thrombopoietin, and vascular endothelial growth factor (VEGF).

### Statistical Analysis

Statistical significance was evaluated using Student’s paired *t*-test using the SIGMASTAT program version 3.0 (SPSS Inc., Chicago, IL, USA). Band densitometric analysis was performed using the Image Processing and Analysis module in Java Program ImageJ (http://rsbweb.nih.gov/ij).

## Results

### IDR-1002 Reduced the LPS-Induced Expression of TNF-α and COX-2

**Figure 1:**
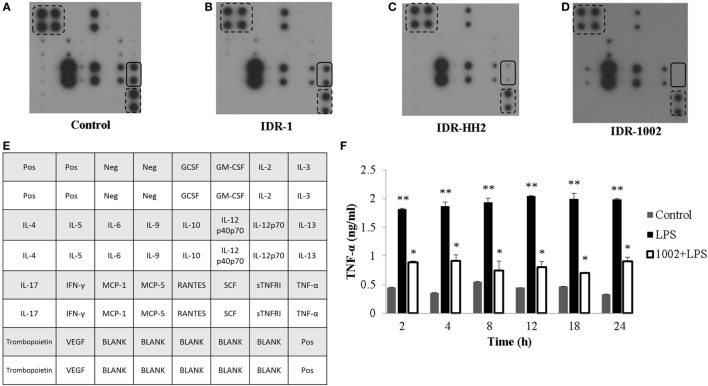
**IDR peptides reduced the expression of TNF-α in RAW 264.7 macrophages**. Macrophages were left unstimulated (control) **(A)** or stimulated with 20 μg/ml of IDR-1 **(B)**, IDR-HH2 **(C)**, or IDR-1002 **(D)** for 12 h. Conditioned media were then collected and centrifuged at 18,800 × *g* for 10 min. The supernatants were transferred to ice-cold tubes and stored at −80°C. Cytokine expression was evaluated with an antibody base-paired array kit (Abcam ab133993), in which antibodies against 22 different cytokines were spotted on a membrane. **(E)** Cytokines array map. In each array, the position of TNF-α is indicated with continuous-line rectangles while positive controls are indicated with dashed-line rectangles. **(F)** Cells were left unstimulated (control), stimulated with 10 ng/ml LPS, or pretreated with 20 μg/ml IDR-1002 for 1 h followed by incubation with 10 ng/ml LPS for 2–24 h. Supernatants were collected and treated as described above. TNF-α quantitation was performed by ELISA (R&D), according to the manufacturer’s instructions. Data represent means ± SEM (*n* = 3). **P < 0.05 for LPS assays compared with the unstimulated control value. *P < 0.05 for 1002 + LPS assays compared with LPS treatment.

To explore the effect of IDR-1, IDR-HH2, and IDR-1002 on cytokine expression, we used an antibody array of 22 cytokines. After 12 h of incubation with each of the IDR peptides, we evaluated the level of cytokine expression in the supernatants. Compared to control (Figure 1A), IDR-1 had a modest effect on TNF-α expression (Figure 1B). However, when macrophages were treated with IDR-HH2 or IDR-1002, the basal levels of TNF-α were significantly reduced. We observed that IDR-1002 was a somewhat stronger inhibitor than IDR-HH2 (Figures 1C,D). A map of the cytokine array is shown in Figure 1E. To determine the kinetics of the effect of IDR-1002 on TNF-α production, macrophages were pre-incubated with IDR-1002 for 1 h and then stimulated, for periods of 2–24 h, with LPS from *E. coli*, an agonist that induced a strong release of TNF-α. We observed that production of TNF-α activated by LPS was strongly reduced in the presence of IDR-1002 (Figure 1F). These results indicate that IDR-1002 had an inhibitory effect on the basal and LPS-induced expression of TNF-α.

**Figure 2:**
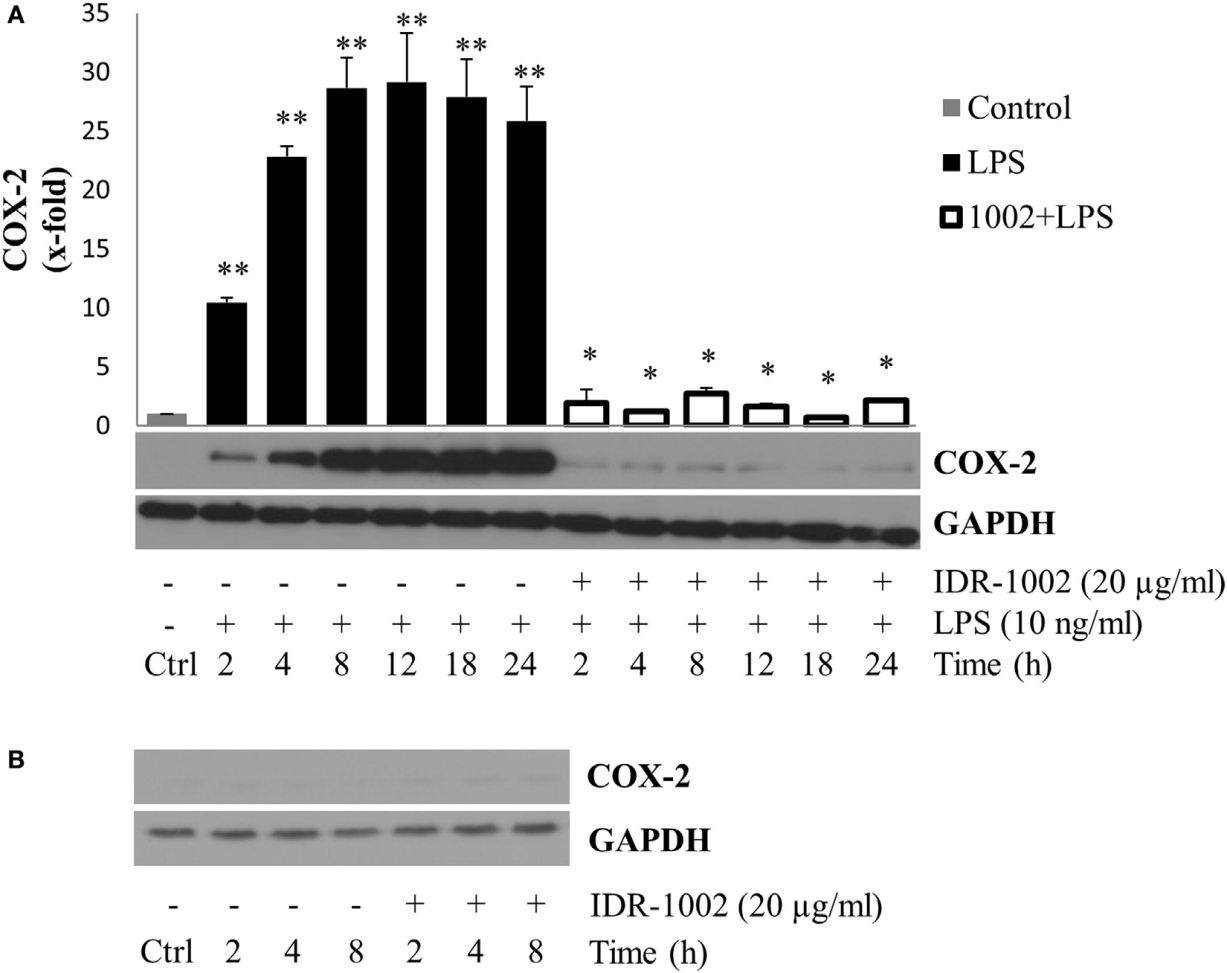
**IDR-1002 inhibited the LPS-induced COX-2 expression in RAW 264.7 macrophages**. **(A)** Macrophages Raw 264.7 were left unstimulated (Ctrl), stimulated with 10 ng/ml of LPS, or pretreated with 20 μg/ml of IDR-1002 for 1 h followed by incubation with 10 ng/ml of LPS for 2–24 h. At the end of each incubation period, COX-2 was analyzed by Western blotting. Protein from Ctrl cells were collected at 4 h. A representative immunoblot of three independent experiments is shown. GAPDH detection was used as control to ensure equal protein loading. **(B)** At each indicated incubation time, COX-2 expression was measured in untreated control and IDR-1002-treated cells. Values were normalized with GAPDH as protein loading control. This graph is representative of two independent experiments. Data represent means ± SEM (*n* = 3). **P < 0.05 for LPS compared with the unstimulated control value. *P < 0.05 for 1002 + LPS compared with LPS treatment.

Another important gene expressed in the inflammatory response and induced by LPS is COX-2. This enzyme catalyzes the conversion of arachidonic acid into prostaglandins and thromboxane, playing an important role in the inflammatory response (34). To determine the effect of IDR-1002 on COX-2 production, macrophages were pre-incubated with IDR-1002 for 1 h and then stimulated with LPS for a period of 2–24 h. Under this condition, we observed that COX-2 expression was strongly and significantly reduced in the presence of IDR-1002 (Figure 2A). Incubation of macrophages with IDR-1002 alone for 2, 4, and 8 h did not have any effect on COX-2 (Figure 2B). These results indicate that IDR-1002 had an inhibitory effect on the LPS-induced expression of COX-2.

### IDR-1002 Reduced the Phosphorylation of NF-κB p65 Subunit at Ser536 and Its Nuclear Translocation

**Figure 3:**
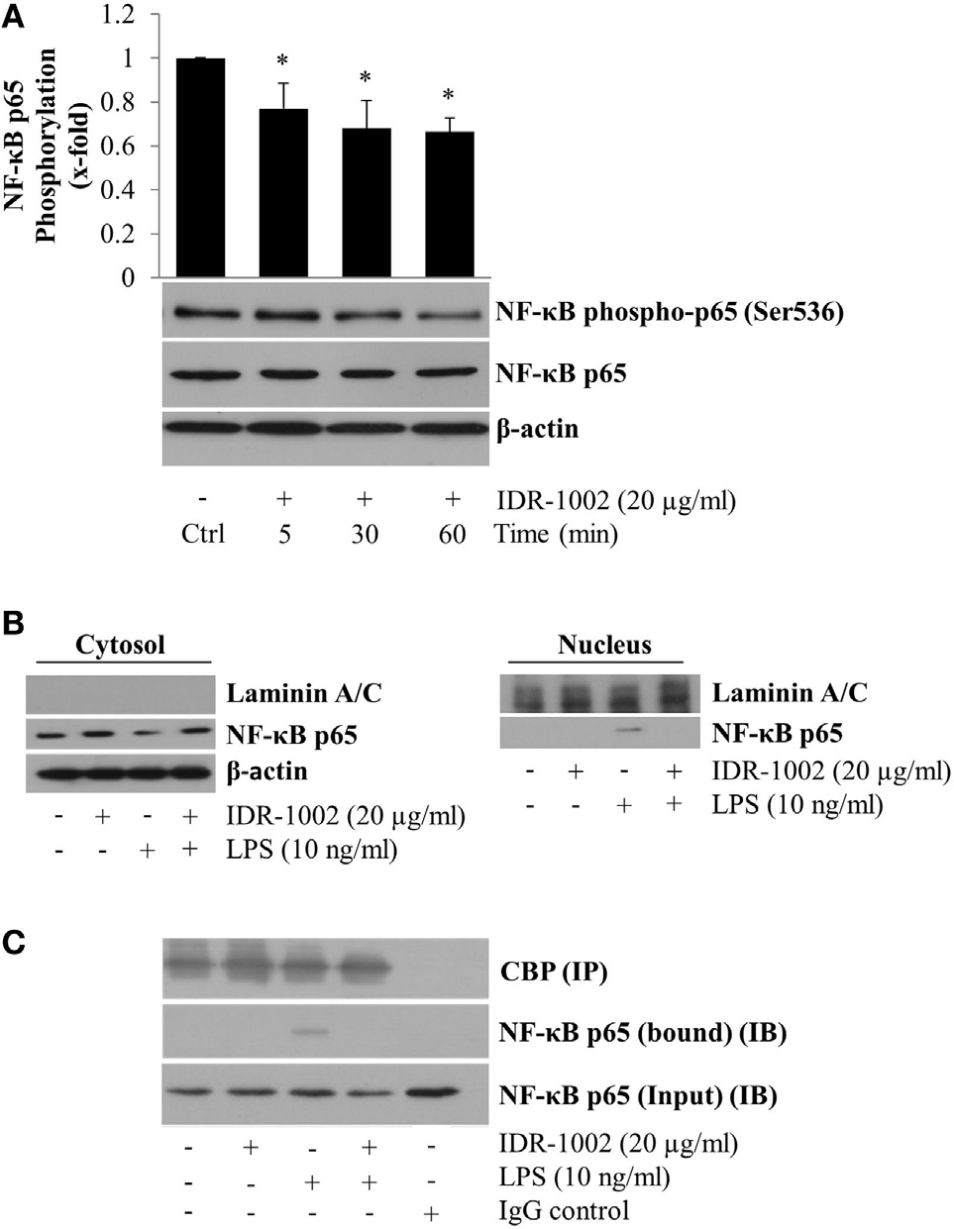
**IDR-1002 reduced NF-κB p65 phosphorylation at Ser536 and inhibited its LPS-activated nuclear translocation in RAW264.7 macrophages**. **(A)** Macrophages were left unstimulated (Ctrl) or stimulated with 20 μg/ml of IDR-1002 for 5–60 min. Phosphorylation of NF-κB p65 subunit was detected by Western blotting. Detection of β-actin and unphosphorylated p65 in each sample was performed to ensure equal protein loading. **(B)** Macrophages were pretreated with 20 μg/ml of IDR-1002 for 1 h and then with 10 ng/ml of LPS for 2 h. Non-phosphorylated NF-κB p65 subunit was detected by Western blotting in nuclear and cytosolic protein enriched fractions. Laminin A/C and β-actin were detected as controls for nuclear and cytosolic fractions, to observe equal protein loading and no cross-contamination between nuclear and cytosolic fractions, respectively. **(C)** Macrophages were treated as described in **(B)**. Eluates from CBP immunoprecipitates (IP) were subjected to Western blot analysis with NF-κB p65 antibody to detect the p65 bound to CBP and its amount present in the input (IB). Control with isotype IgG was also included. Detection of CBP was performed to show equal protein loading. *P < 0.05 for IDR-1002 compared with the unstimulated control value.

Activation of NF-κB stimulates its nuclear translocation leading to the expression of many pro-inflammatory genes, among which TNF-α and COX-2 play important roles in the inflammatory response. To explore if IDR-1002 inhibited TNF-α and COX-2 expression by inhibiting NF-κB activity, we evaluated phosphorylation of the p65 subunit at Ser536 (a residue located in the transactivation domain), and its nuclear relative abundance. An ~20–40% reduction of p65 phosphorylation at Ser536 was observed when macrophages were incubated with IDR-1002 for 5, 30, or 60 min (Figure 3A). Next, we investigated the relative abundance of p65 in the cytoplasm and nucleus of macrophages incubated with LPS and IDR-1002. From data shown in Figure 3B, it was clear that LPS stimulated p65 nuclear translocation, which was inhibited by IDR-1002. The co-immunoprecipitation of NF-κB and coactivator CBP was detected exclusively in macrophages stimulated with LPS, but not in those stimulated with IDR-1002 followed by LPS or IDR-1002 alone (Figure 3C). These results indicate that IDR-1002 reduced the relative abundance of p65 phosphorylated at Ser536 and its LPS-induced nuclear translocation and interaction with CBP.

### IDR-1002 Inhibited the Phosphorylation and Degradation of IkBα Induced by LPS

**Figure 4:**
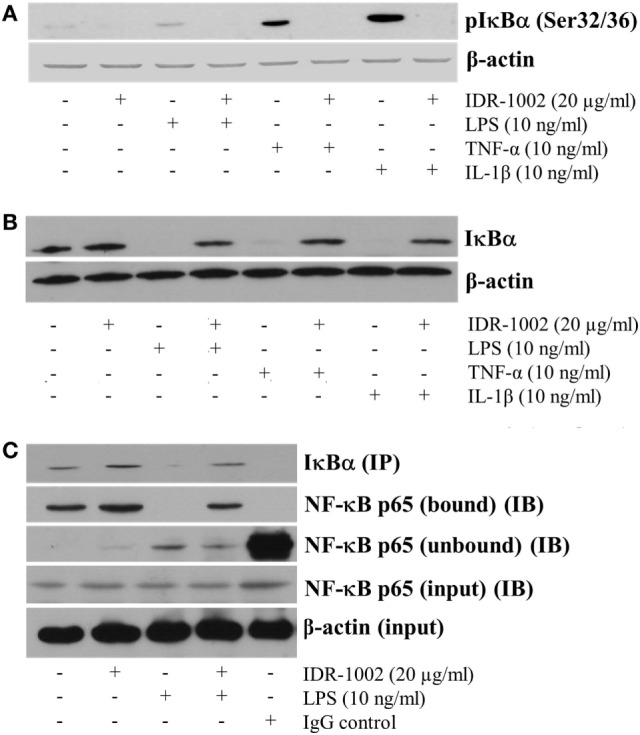
**IDR-1002 inhibited IκBα phosphorylation and degradation in RAW 264.7 macrophages**. **(A)** Macrophages were left unstimulated or pretreated with 20 μg/ml of IDR-1002 for 1 h. Then, macrophages were stimulated for 10 min with either 10 ng/ml of LPS, 10 ng/ml of TNF-α, or 10 ng/ml of IL-1β. Phosphorylated IκBα was detected by Western blot analysis and β-actin was detected to ensure equal protein loading. **(B)** Macrophages were treated as in **(A)** except that incubation with LPS, TNF-α, or IL-1β was for 30 min. Total IκBα was detected by Western blot analysis in total cell lysates extracts. β-actin detection was performed to ensure total protein loading. **(C)** Macrophages were left unstimulated or stimulated with 20 μg/ml of IDR-1002 for 1 h followed by stimulation with 10 ng/ml of LPS for 10 min. IκBα was immunoprecipitated (IP) and eluates were subjected to Western blot to detect NF-κB p65 in the immunoprecipitates of IκBα (bound) or supernatants (unbound) samples (IB). β-actin detection was performed in the input cell extracts to ensure equal protein.

In unstimulated cells, NF-κB is anchored to IκBα in the cytoplasm. Release of NF-κB requires phosphorylation of IκBα, which marks it to become ubiquitinated and then degraded in the proteosome. This mechanism implies that if phosphorylation of IκBα is inhibited, then NF-κB would not be released and its nuclear translocation would not occur. Our data indicate that IDR-1002 strongly inhibited the LPS-induced phosphorylation of IκBα (Figure 4A) and its degradation (Figure 4B). This effect of IDR-1002 on IκBα seemed to be independent of the stimulus because inhibition of IκBα phosphorylation and degradation by IDR-1002 was equally effective in macrophages stimulated with TNF-α or IL-1β (Figures 4A,B). Interaction of NF-κB p65 and IκBα was stabilized as a consequence of IDR-1002 inhibition of IκBα phosphorylation and degradation. Figure 4C shows that there was an increase in co-immunoprecipitation NF-κB p65 bound to IκBα in macrophages stimulated with IDR-1002. These data may explain the inhibitory effect exerted by IDR-1002 on TNF-α and COX-2 expression.

### Activation of CREB Phosphorylation at Ser133 by IDR-1002 Was Dependent on ERK1/2, p38, and MSK1 Activity

**Figure 5:**
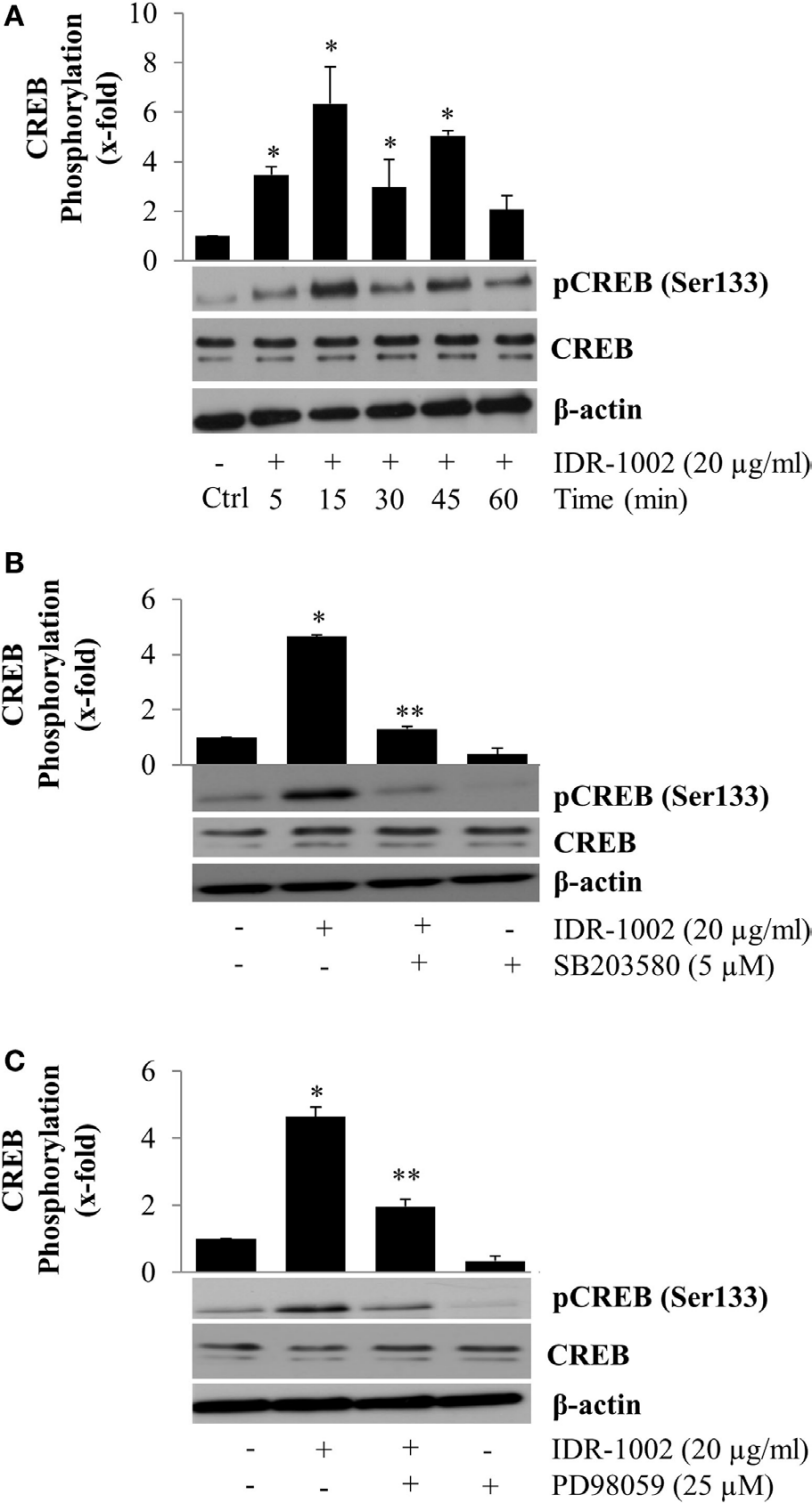
**Inhibition of p38 and ERK1/2 activity reduces CREB phosphorylation at Ser133 in Raw 264.7 macrophages activated by IDR-1002**. **(A)** Macrophages were left unstimulated (Ctrl) or stimulated with 20 μg/ml of IDR-1002 for 5–60 min. After stimulation, CREB phosphorylation was detected by Western blotting. **(B)** Cells were left untreated (−) or treated with 5 μM of SB203580 (SB, a p38 inhibitor) for 30 min and then stimulated with 20 μg/ml of IDR-1002 for 15 min. **(C)** Cells were left untreated (−) or treated with 25 μM of PD98059 (PD, an ERK1/2 inhibitor) for 30 min and then stimulated with 20 μg/ml of IDR-1002 for 15 min. Additional controls in which cells were treated with 5 μM of SB or 25 μM of PD alone were also included. Phosphorylation of CREB was analyzed by Western blotting. Detection of β-actin and unphosphorylated CREB were performed to ensure equal protein loading. Blots are representative of three independent experiments. The data represent means ± SEM (*n* = 3). *P < 0.05 for IDR-1002 compared with the unstimulated control value. **P < 0.05 for inhibitor + IDR-1002 assays compared with the IDR-1002 assay.

A major mechanism for controlling the inflammatory response is the suppression of pro-inflammatory cytokine expression, which can be promoted when NF-κB forms a molecular complex with CBP (16, 17). This process takes place during the inflammatory response when CREB is also activated by phosphorylation at Ser133. Phosphorylation of CREB enables it to compete with NF-κB for the same interaction domain on CBP (18). The consequence of NF-κB separation from CBP is a reduction in inflammatory cytokine expression and simultaneously an increment, in many but not all cases, of anti-inflammatory cytokine expression due to CREB activity. To explore if IDR-1002 activated CREB phosphorylation at Ser133, macrophages were incubated with IDR-1002 for 5– 60 min. Under these conditions, a significant increase in CREB phosphorylation was observed for up to 45 min of incubation with IDR-1002 (Figure 5A). Because CREB phosphorylation can be mediated by Akt (PKB), ERK1/2, p38, and MSK1/2, we performed experiments utilizing specific inhibitors for each one of these enzymes. Macrophages stimulated with IDR-1002 showed an increased relative abundance of CREB phosphorylated at Ser133 and this was reduced by specific inhibitors of p38 and ERK1/2 (Figures 5B,C). Correspondingly, IDR-1002 induced a strong phosphorylation of ERK1/2 and p38 (Figures S1A,B in Supplementary Material), as described previously (27). However, specific inhibitors of PI3K, GSK3, and Akt did not have any effect on CREB phosphorylation (Figures S2A,B in Supplementary Material).

**Figure 6:**
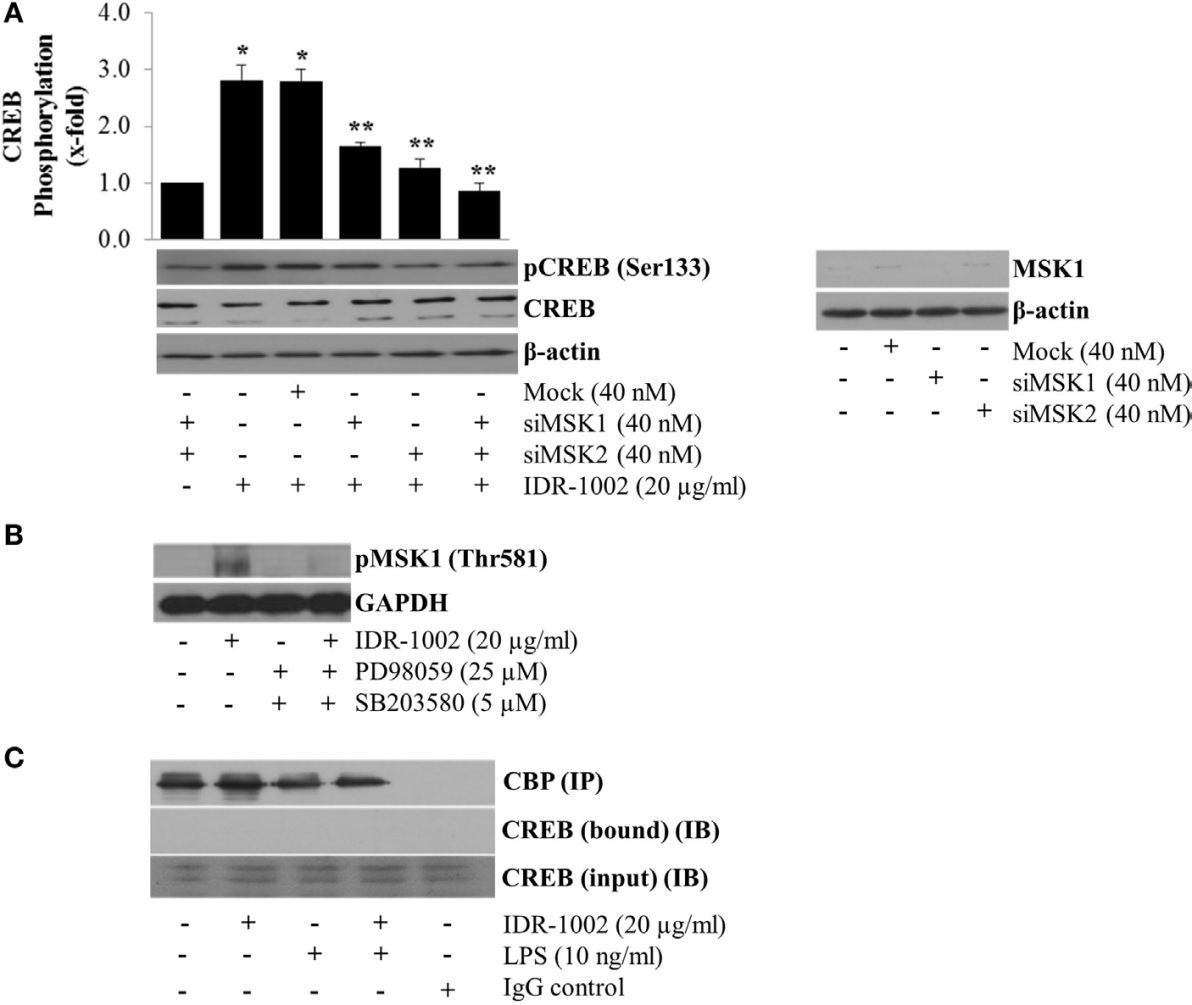
**MSK1-mediated CREB phosphorylation at Ser133 in RAW 264.7 macrophages stimulated with IDR-1002**. **(A)** Macrophages were transfected with non-specific siRNA control (Mock), MSK1 (siMSK1), MSK2 (siMSK2) or MSK1, and MSK2 simultaneously for 48 h. Cells were then stimulated with 20 μg/ml of IDR1002 for 15 min. CREB phosphorylation was analyzed by Western blotting. Detection of β-actin and unphosphorylated CREB in each sample was performed to ensure equal protein loading. The right panel shows detection of MSK1 gene expression when macrophages were treated with MSK1-specific siRNA. In **(B)**, macrophages were left unstimulated (−) or pretreated with 25 μM of PD98059 (PD, an ERK1/2 inhibitor) and 5 μM of SB203580 (SB, a p38 inhibitor) for 30 min, and then stimulated with IDR-1002 for 15 min. Additional controls with 25 μM of PD and 5 μM of SB alone were also included. Under these conditions, phosphorylation of MSK1 was detected by Western blotting. **(C)** Macrophages were left unstimulated or stimulated with 20 μg/ml of IDR-1002 followed by stimulation with 10 ng/ml of LPS. Immunoprecipitation was performed as previously described and eluates from CBP immunoprecipitates (IP) were subjected to Western blot analysis with CREB antibodies. Then, we detected CREB immunoprecipitated (bound) with CBP and in the input (IB). Control with isotype IgG was also included. Detection of CBP was performed to show equal protein loading. The blots are representative of three independent experiments. Data represent means ± SEM (*n* = 3). *P < 0.05, compared with the unstimulated control value. **P < 0.05 for siRNA treatment compared with the IDR-1002 value.

Other authors have reported that ERK1/2 and p38 do not directly phosphorylate CREB but instead they first phosphorylate MSK1/2, which in turn phosphorylates CREB at Ser133 (35). To test if MSK1/2 linked the activation of ERK1/2 and p38 with CREB phosphorylation, we silenced the MSK1 and MSK2 genes using siRNA. When these genes were independently or simultaneously silenced, a significant reduction in CREB phosphorylation was observed (Figure 6A), which indicated that both MSK1 and MSK2 could participate. However, only MSK1 (Figure 6B), but not MSK2 (data not shown), phosphorylation was detected in macrophages stimulated with IDR-1002. Evidence of MSK1 gene knockout by siRNA is presented in Figure 6A. Furthermore, incubation of macrophages with either ERK1/2- or p38-specific inhibitors almost completely prevented phosphorylation of MSK1 (Figure 6B).

However, although CREB was strongly phosphorylated, we observed no increase in the anti-inflammatory cytokines IL-4, IL-10, and IL-13 as measured by ELISA (data not shown). The lack of expression of these cytokines associated with M2 macrophage response might be due to the absence of interaction between phosphorylated CREB and CBP. The co-immunoprecipitation of CREB with CBP from macrophages stimulated with IDR-1002 showed that no complex was formed between CBP and CREB (Figure 6C). Together, these genetic and biochemical data indicated that IDR-1002-induced phosphorylation of CREB at Ser133 depended on the ERK1/2/p38–MSK1 signaling pathway, and that, at least in our system, phosphorylation of CREB by MSK1 did not promote interaction between CREB and CBP.

## Discussion

Data presented here demonstrated that in RAW264.7 macrophages/monocytes stimulated with LPS, IDR-1002 was able to inhibit IκBα phosphorylation and degradation, completely blocking NF-κB p65 nuclear translocation. This would explain the inhibition of the interaction between NF-κB and CBP, and partially inhibition of NF-κB p65 Ser536-phosphorylation, which can enhance transactivation. These effects of IDR-1002 on NF-κB p65, together with others previously reported (36, 37), were sufficient to reduce the expression of TNF-α and COX-2. In addition, IDR-1002 induced a significant increase in CREB phosphorylation at Ser133 through the activation of p38/ERK1/2 and MSK1/2. Although the relative abundance of phosphorylated CREB was higher in macrophages stimulated with IDR-1002 than in untreated controls, no interaction between CREB and CBP occurred, and thereby preventing the enhanced expression of cytokines IL-4, IL-10, and IL-13.

Inhibition of TNF-α expression in RAW264.7 macrophages has been demonstrated by *in vitro* administration of different compounds in response to LPS from *E. coli* (38–41). For example, the anti-inflammatory effect of extracellular phospho-ceramide analog-1 (PCERA-1) was found to depend on suppression of TNF-α production, an increase in cAMP levels, and increased expression of IL-10 (38). The low molecular weight chemical compounds madecassic and asiatic acid, and the isoflavone genistein potently inhibited the expression of TNF-α, COX-2, NO, prostaglandin E_2_, IL-1β, and IL-6 by blocking both IκBα degradation and NF-κB nuclear translocation (39–41). The effects in macrophages induced by IDR-1002 on TNF-α/COX-2 expression, and NF-κB inhibition, resemble those triggered by the compounds mentioned above, suggesting that parallel mechanisms mediate the effect of IDR-1002 to control inflammation. Specifically, we found in RAW 264.7 macrophages that IDR-1002 was able to inhibit LPS-induced phosphorylation of IKKα and IKKβ, which may explain the inhibitory effect of this peptide on IκBα phosphorylation (Figure S3 in Supplementary Material). Recently, Liu et al. (42) discovered that a long non-coding RNA inhibits IκB phosphorylation by forming a ternary complex with NF-κB and IκBα, which caused a strong inhibition of metastasis in breast cancer. Based on these results, it is possible that IDR-1002, as an inhibitor of IKK and IκBα phosphorylation, may be a good candidate for metastasizing cancers. We are currently performing experiments to test IDR-1002 in various cancer cell lines.

COX-2 is important in the control of inflammation, pain, fever, and other physiological processes. Inhibition of COX-2 activity by non-steroidal anti-inflammatory drugs (NSAIDs) such as aspirin has been the clinicians’ choice to alleviate medical conditions. However, NSAIDs induce tolerance over time (43) and administration for even short periods can cause gastrointestinal and renal secondary effects in ~25% of individuals and almost 5% of them suffer serious health problems (44). Because of these detrimental effects induced by NSAIDs, a search for new anti-inflammatory drugs without side effects is of primary importance in the pharmaceutical industry. The strong inhibition by IDR-1002 of LPS-induced COX-2 expression observed in our study suggests that IDR peptides may be a viable alternative. However, more experiments *in vivo* and chemical modification/formulation of IDR-1002 to increase its potency, specificity, and half-life time must be considered before its administration to human beings.

Apart from the inhibitory effect of IDR-1002 on LPS-induced NF-κB and COX-2, this cationic peptide induced the phosphorylation/activation of p38, ERK1/2, and MSK1, which led to the phosphorylation of CREB at its Ser133 activation site. The contribution of the signaling pathway PI3K/Akt/GSK3 as an effector of IDR-1002-induced CREB phosphorylation was ruled out (Figure S2 in Supplementary Material). According to one current model, once CREB becomes phosphorylated at Ser133, it is able to interact with CBP and induce expression of anti-inflammatory cytokine genes, among which IL-4, IL-10, and IL-13 are the most important for reducing inflammation (23). However, it has been documented that in some cases phosphorylation of CREB at Ser133 by MSK1/2 is not absolutely required to promote recruitment of CBP and, consequently, expression of some CREB target genes does not take place (15). Similarly, activation of T-cell receptor (TCR) promotes a strong phosphorylation of CREB at Ser133 without formation of CREB–CBP complex, unless low amounts of cAMP were present as a costimulus (24). In our case, IDR-1002-activated MSK1-dependent phosphorylation of CREB did not promote the interaction of CREB with CBP, which might explain lack of effects on gene expression of IL-4, IL-10, and IL-13. This conclusion was drawn after several attempts to co-immunoprecipitate CBP with CREB in protein extracts recovered from macrophages stimulated with IDR-1002. More experiments to find out the molecular details of this mechanism are currently underway in our laboratory.

The experimental evidence obtained in RAW 264.7 macrophages indicating that IDR-1002 inhibits LPS-induced COX-2 expression, activates CREB phosphorylation at Ser133, and inhibits LPS-induced IκBα degradation was confirmed in PBMC (Figures S4A–C in Supplementary Material). This novel spatiotemporal combination of both effects (inhibition of NF-κB signaling and activation of CREB phosphorylation) makes IDR-1002 an attractive anti-inflammatory pharmaceutical drug.

## Author Contributions

AH-M performed most of the experiments and helped with the writing of the manuscript. OS-G performed many experiments. JO-B helped to perform some experiments. RH financially supported the research (provided the peptide IDR-1002) and critically reviewed the manuscript. VMB-A financially supported most of the supplies and wrote the manuscript.

## Conflict of Interest Statement

The authors declare that the research was conducted in the absence of any commercial or financial relationships that could be construed as a potential conflict of interest.
