# Supplementary figures and images for: Peptide IDR-1002 Inhibits NF-κB Nuclear Translocation by Inhibition of IκBα Degradation and Activates p38/ERK1/2–MSK1-Dependent CREB Phosphorylation in Macrophages Stimulated with Lipopolysaccharide
Source: Front Immunol. 2016 Nov 25;7:533. doi: 10.3389/fimmu.2016.00533 (PMC5122595; doi:10.3389/fimmu.2016.00533)

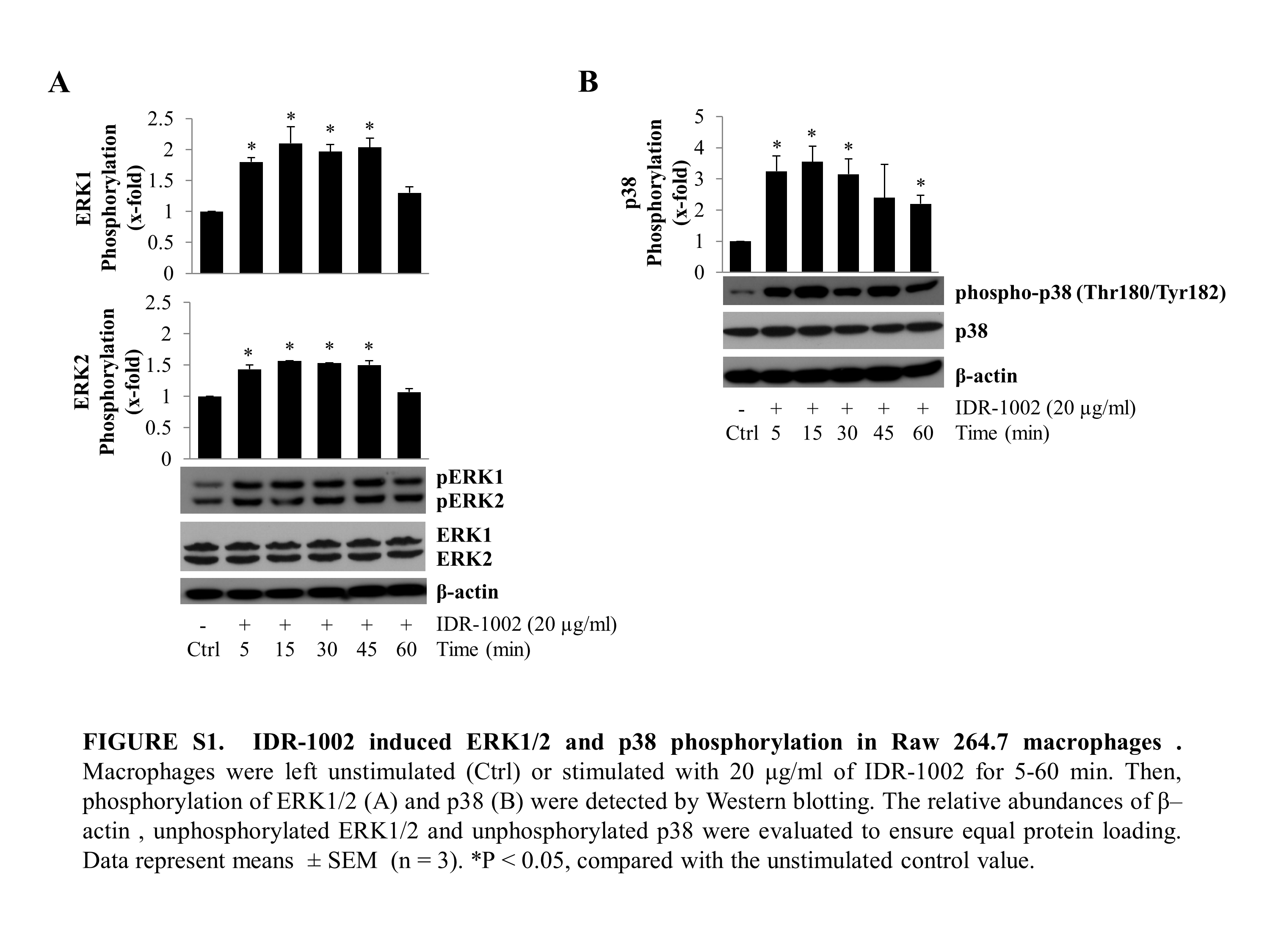

Supplement: Supplementary file 1 [file Figure_7.tif]

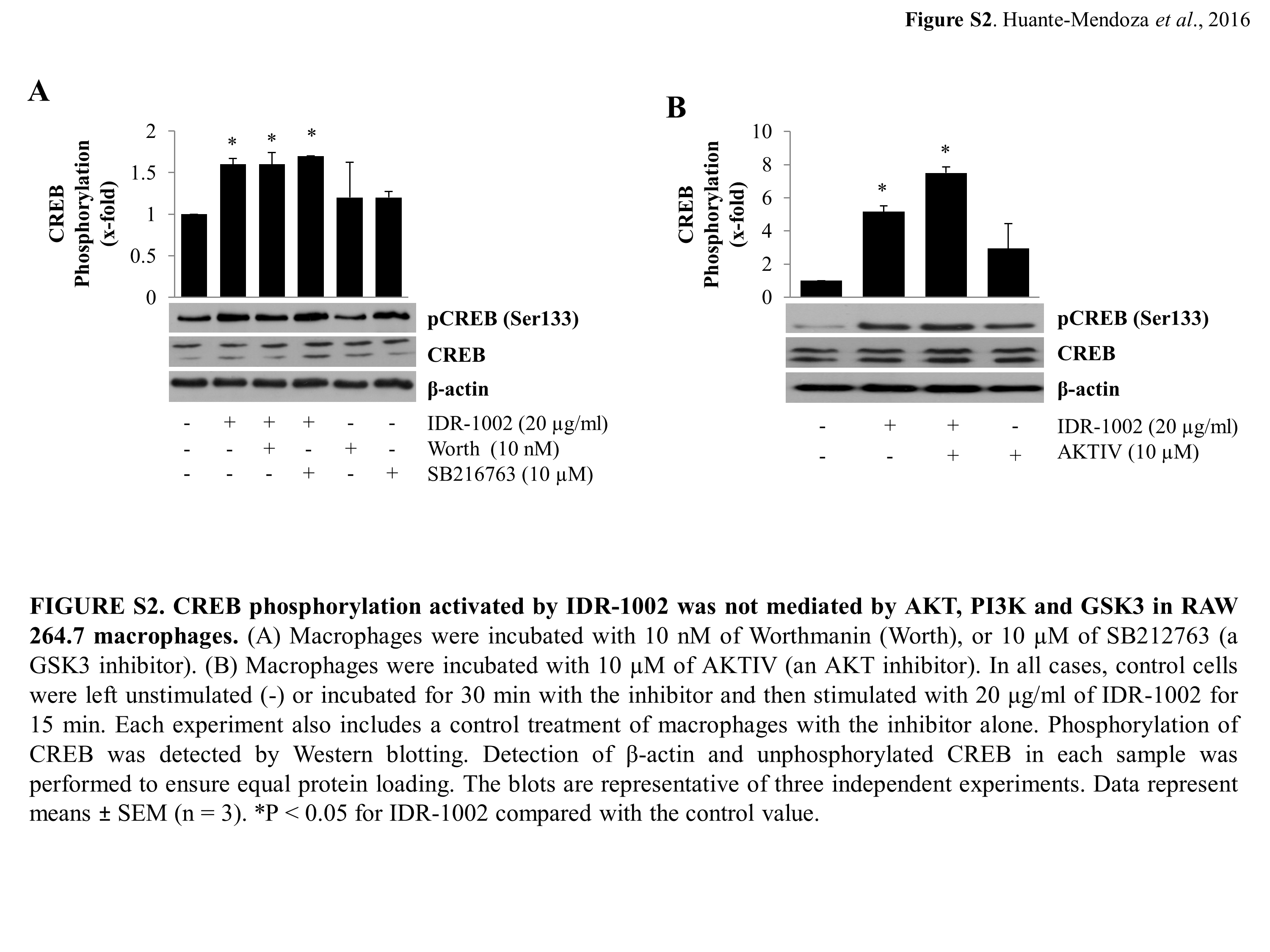

Supplement: Supplementary file 2 [file Figure_8.tif]

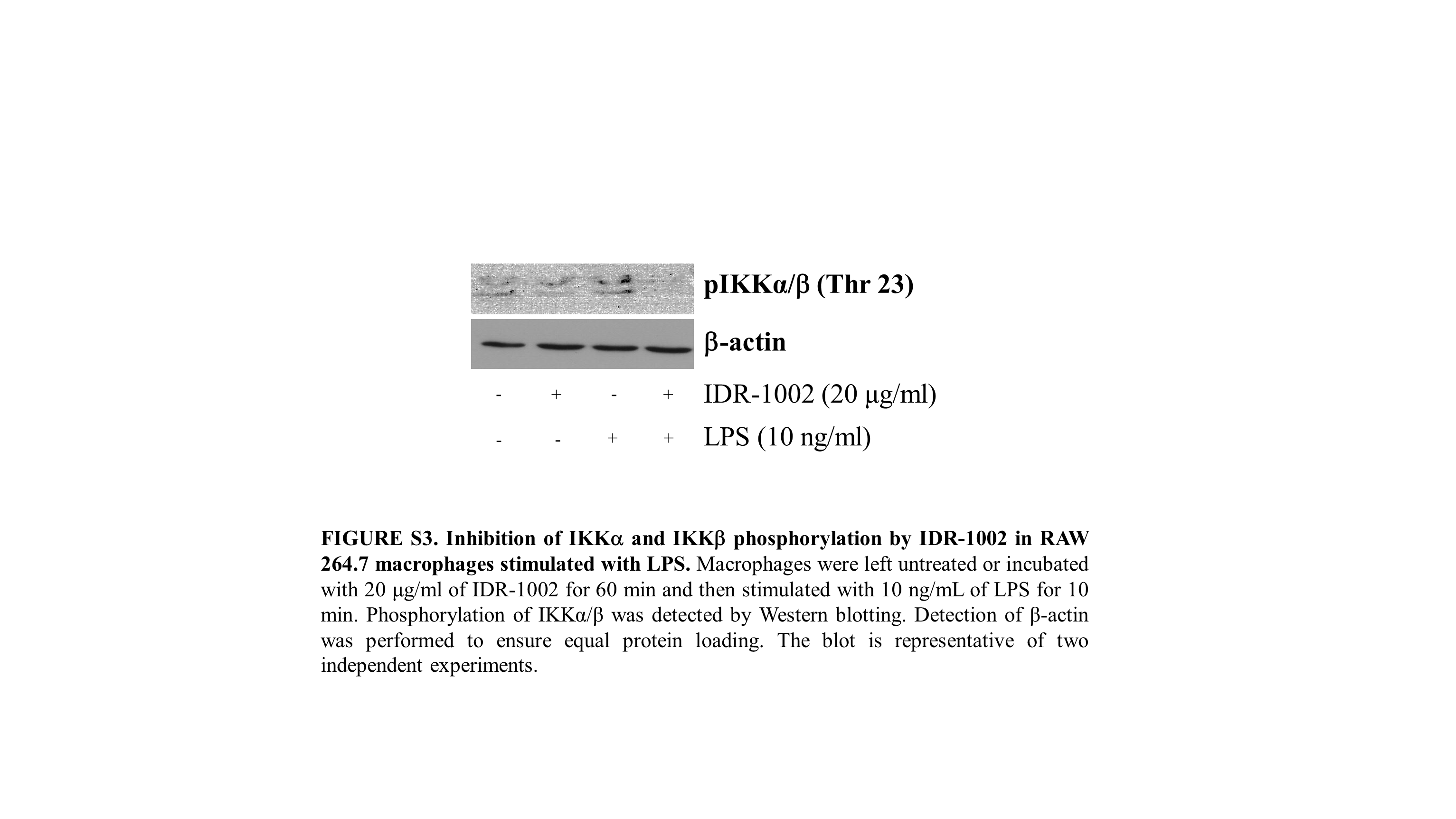

Supplement: Supplementary file 3 [file Figure_9.tif]

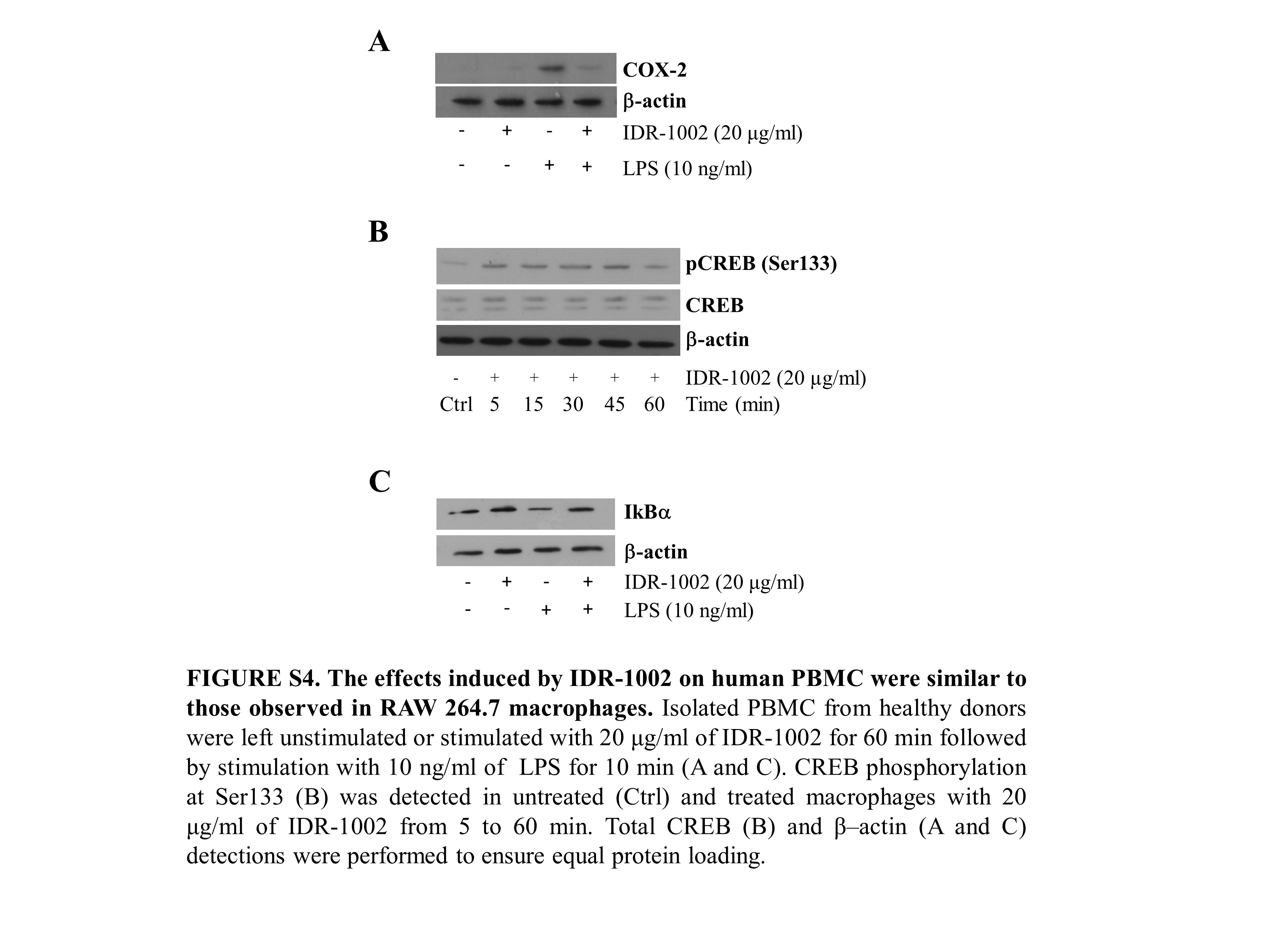

Supplement: Supplementary file 4 [file Figure_10.tif]
